# Broadcasting revenue sharing after cancelling sports competitions

**DOI:** 10.1007/s10479-023-05246-0

**Published:** 2023-03-14

**Authors:** Gustavo Bergantiños, Juan D. Moreno-Ternero

**Affiliations:** 1 ECOBAS, Universidade de Vigo, ECOSOT, 36310 Vigo, Spain; 2 Department of Economics, Universidad Pablo de Olavide, 41013 Sevilla, Spain

**Keywords:** Game theory, Resource allocation, Broadcasting, Cancelled seasons, C71, D63, Z20

## Abstract

The COVID-19 pandemic forced the partial or total cancellation of most sports competitions worldwide. Sports organizations crucially rely on revenues raised from broadcasting. How should the allocation of these revenues be modified when sports leagues are cancelled? We aim to answer that question in this paper by means of the axiomatic approach. Two extension operators (dubbed *zero* and *leg* operators, respectively) will play a major role in our analysis. We show that several combinations of axioms (formalizing ethical or strategic principles) characterize the image via those operators of two focal rules: the * equal-split* rule and *concede-and-divide*.

## Introduction

Over the past years, professional sports leagues have gained momentum with an increasing number of fans paying to watch games on television. According to Statista, in 2019, the NFL’s broadcasting rights amounted to 4.4 billion U.S. dollars domestically and 120 million U.S. dollars overseas. The English Premier League raised 2.08 billion U.S. dollars domestically and 1.75 billion U.S. dollars abroad. It can be safely argued that the sale of broadcasting rights is currently the biggest source of revenue for sports teams.

Most of the sports broadcasting contracts have recently been in jeopardy. The COVID-19 pandemic caused major lockdowns worldwide, confining all the population and maintaining only essential services, for non-negligible periods of time. Among many other things, this forced the partial or total cancellation of an endless list of sports competitions.^1^ Most important competitions resumed after lockdowns (in spite of having empty stadiums) to secure broadcasting contracts. But some others did not, and ended up cancelling the season. It was the case of the football leagues in France, the Netherlands and Belgium, among others. We are concerned in this paper with the renegotiation of those broadcasting contracts after cancelling sports seasons.

The sale of broadcasting rights for sports leagues is often carried out collectively. Afterwards, the revenues collected from the sale have to be shared, which becomes a crucial aspect for the management of sports organizations. We recently introduced a simple formal model (Bergantiños & Moreno-Ternero, 2020a) in which the sharing process is based on the (broadcasting) audiences that the games throughout the season generate. We considered two simplifying assumptions: a double round-robin tournament (with a fixed set of teams), in which all games had a constant pay-per view fee. Thus, the prior of the model was simply a square matrix, whose entries indicate the audience of the game involving the row team and the column team, at the former’s stadium. We extend that model here to account for cancelled seasons.^2^ More precisely, the extended model allows for empty (to be distinguished from zero) entries in the matrix and it also considers an external endowment to be allocated (thus avoiding the simplifying assumption of constant pay-per view fee).

A natural way to address the extended model would be to ignore the nature of cancelled games, just treating them as games with zero audience, and solve the allocation problem with the actual (lower) endowment after cancellation. But this might be unfair for (weaker) teams whose games with popular teams were cancelled. For instance, in the case of Ligue 1 mentioned above, Nimes was supposed to play PSG in late May 2020, and the game was cancelled, whereas Amiens was able to play PSG in mid February 2020, weeks before confinement measures took place. Thus, assigning Nimes zero audience in such a game would hurt it in the allocation process with respect to Amiens, which was lucky to play the audience-boost PSG game. Prompted by this case, we shall also explore an alternative way to address the extended model: assigning to cancelled games the audience of the corresponding game in the first leg of the tournament, provided this was not cancelled.

More precisely, we construct two operators that convert audience matrices for cancelled leagues into audience matrices for (fictional) non-cancelled leagues. Both operators leave audiences of non-cancelled games unchanged. Thus, non-empty entries remain the same. And they treat empty entries differently. The *zero operator* converts them into zero. The * leg operator* converts them into their symmetric entries (provided they are not empty). If we consider benchmark rules that solve the allocation problem for non-cancelled leagues, then the operators convert them into rules that solve the allocation problem for cancelled leagues. We then take the axiomatic approach to explore the two routes that the previous two operators convey. We show that several combinations of natural axioms characterize the extensions of two focal rules from the benchmark model: the * equal-split* rule and *concede-and-divide* (Bergantiños & Moreno-Ternero, 2020a).

We conclude this introduction mentioning that the UEFA Champions League will see a transformation from the traditional group stage to a single league phase including all participating teams from the 2024/25 season (UEFA, 2022). Each of the 36 clubs will play 8 games against 8 different opponents (four home games, four away). Thus, we can consider this phase as a round-robin tournament where many games have been cancelled (the ones that have not been played because of the new format). Our model could also be applied to study this new tournament format.

### Related literature

The broadcasting problem we consider here is a specific resource allocation problem, akin to well-known problems largely analyzed in the literature. Instances are land division (e.g. Steinhaus, 1948; Chambers, 2005), claims problems (e.g. O’Neill, 1982; Thomson, 2019), telecommunications problems (e.g. van den Nouweland et al., 1996; Altman et al., 2006), museum pass problems (e.g. Ginsburgh and Zang, 2003; Bergantiños and Moreno-Ternero, 2015), or cost sharing associated with classical operations research problems, such as the minimum cost spanning tree problem (e.g. Bergantiños and Vidal-Puga, 2007; Bergantiños and Lorenzo, 2021) or the job scheduling problem (e.g. Gertsbakh and Stern, 1978; Bahel and Trudeau, 2019).

The benchmark model (with no cancelled games) has been studied in several papers. In Bergantiños and Moreno-Ternero (2020a), the formal model is introduced, and two sharing rules (*equal-split* and * concede-and-divide*) are considered. Besides, the theoretical results are applied to La Liga (the Spanish football league). In Bergantiños and Moreno-Ternero (2020b), those two rules (as well as the *uniform* rule) are characterized via axioms that formalize the principle of * marginalism*. In Bergantiños and Moreno-Ternero (2021), the family of rules obtained as convex combinations of *equal-split* and * concede-and-divide* is explored. It is argued that this family is quite relevant from a practical point of view, as applied to La Liga.^3^ In Bergantiños and Moreno-Ternero (2022a, 2022b), the axiomatic approach is further explored, with a special emphasis on alternative axioms formalizing the principle of *impartiality*, which leads to characterizations of alternative families of rules compromising among some of the focal rules mentioned above. In Bergantiños and Moreno-Ternero (2022b), the focus is on *monotonicity* axioms, which have a long tradition in axiomatic work and allow us to uncover further the structure of the benchmark model. In Bergantiños and Moreno-Ternero (2022c), we obtain the most general characterization in the benchmark model, which provides normative foundations for a large family of rules encompassing all the rules that had been independently characterized in the benchmark model so far. Finally, in Bergantiños and Moreno-Ternero (2023a) we explore a decentralized approach to select, via majority voting, among the existing rules in the literature.

To conclude, we stress that the allocation of broadcasting revenues from a cancelled season is not necessarily independent of ranking the teams on the basis of the games that have been played. This ranking problem is precisely what Csató (2021a, 2021b), Van Eetvelde et al. (2021), and Gorgi et al. (2021) studied recently. The approach of Csató (2021a) is actually similar to ours. He proposes a ranking (for a cancelled season) and uses the axiomatic approach to justify it. Similarly, attention has recently been given to the axiomatic approach to the somewhat related problem of prize/revenue allocation in sports competitions (e.g., Petróczy and Csató, 2021; Dietzenbacher and Kondratev, 2022). Finally, Csató (2022b) demonstrates through a real world example why monotonicity is an important property of revenue distribution systems.

### Preview of our results

As mentioned above, we take the axiomatic approach to deal with our problem. To wit, we explore the implications of several basic axioms for allocation rules.

The first two axioms we consider are natural extensions of two axioms from the benchmark model. *Null team on non-cancelled games* says that if the audience of each game played by a team is zero, then this team obtains no revenue. *Essential team on non-cancelled games* says that if all games with positive audience are played by a team, then this team obtains all the revenue.

The rest of the axioms we consider are new.

*Baseline monotonicity* compares the allocation in two situations that arise after modifying the audience of a specific game, say played by *i* and *j* at *i*’s stadium. If the audience has increased, *baseline monotonicity* says that teams *i* and *j* could not receive less whereas the rest of the teams could not receive more, provided that the total revenue is the same in both situations. If the game has only been played at the second situation, then we apply the same idea, but now comparing the audience of that game with the audience given by the operator to the cancelled game in the first situation.

*Reallocation proofness* compares two tournaments where the aggregate audience of a given team, as well as the aggregate audience, coincide. The axiom says that this team should receive the same in both tournaments.

The last axioms we consider assume leagues are divided into conferences. Suppose that, for such a framework, only games among teams in the same conference have a positive audience. Then, instead of solving the whole tournament, we can solve each conference tournament separately, assuming that the revenue is divided among the conference tournaments proportionally to their estimated audiences, computed via the operator. We consider two axioms, depending on how we define the conference tournament. In the * single-conference* axiom, each team plays a single tournament (the one given by the teams of its conference). In the *multi-conference* axiom, each team plays several tournaments. For each conference, we consider a tournament in which all teams participate, but only the games involving the teams within the conference have been played.

In the benchmark model (Bergantiños & Moreno-Ternero, 2020a) two main rules arise. The *equal-split* rule splits the revenue generated from each game equally among the participating teams, whereas * concede-and-divide* concedes each team the revenues generated from its fan base and divides the residual equally. We show that the *extended equal-split rule via the zero operator* is characterized by two combinations of axioms. First, by *reallocation proofness* and * single-conference*. Second, by *reallocation proofness*, *null team for non-cancelled games* and *multi-conference*. We also show that the *extended equal-split rule via the leg operator* is also characterized by two combinations of axioms. First, by *weak reallocation proofness* (that is, requiring *reallocation proofness* only for tournaments in which no game has been cancelled), * single-conference*, and *baseline monotonicity.* Second, by * weak reallocation proofness*, *baseline monotonicity*, *null team for non-cancelled games* and *multi-conference*. Finally, we show that the *extended concede-and-divide via the zero operator* is characterized by *reallocation proofness*, *essential team on non-cancelled games*, and *multi-conference*. And that the * extended concede-and-divide via the leg operator* is characterized by *weak reallocation proofness*, *essential team on non-cancelled games*, *multi-conference*, and *baseline monotonicity*.

If we restrict to the benchmark model we also obtain new characterizations of the *equal-split* rule and *concede-and-divide*. Thus, our results can not be seen as generalizations of results from the benchmark model to our setting.

The rest of the paper is organized as follows. In Sect. 2, we introduce the model (benchmark rules, operators, extended rules and axioms) together with some illustrative examples to motivate our problem further. In Sect. 3, we provide the characterization results. Section 4 concludes. For a smooth passage, proofs are deferred to an appendix.

## The model

Let *N* be a finite set of teams. Without loss of generality, we usually take $N=\{1,2,\dots ,n\}$. We assume $n\ge 4$. We also assume a round-robin tournament in which each team plays in turn against each other team twice (home and away), which is the typical format of many national football leagues.^4^ For each pair of teams $i,j\in N$, we denote by $a_{ij}$ the broadcasting audience (number of viewers) for the game played by *i* and *j* at *i*’s stadium. We write $a_{ij}=\varnothing$ if the game was cancelled. For notational simplicity, we also assume that $a_{ii}=\varnothing$ for each $i\in N$. Let $A\in {\mathcal {A}}_{n\times n}$ denote the resulting matrix of broadcasting audiences generated in the whole tournament involving the teams within *N*.

Let $\alpha _{i}\left( A\right)$ denote the total audience achieved by team *i*, i.e.,

$$\begin{aligned} \alpha _{i}\left( A\right) =\sum \limits _{i\in \left\{ j,k\right\} \subset N,a_{jk}\ne \varnothing }a_{jk}. \end{aligned}$$

We take $\alpha _{i}\left( A\right) =0$ when team *i* has not played any game. When no confusion arises, we write $\alpha _{i}$ instead of $\alpha _{i}\left( A\right) .$

For each $A\in {\mathcal {A}}_{n\times n}$, let ||*A*|| denote the total audience of the tournament. Namely,

$$\begin{aligned} ||A||=\sum _{i,j\in N,a_{ij}\ne \varnothing }a_{ij}=\frac{1}{2}\sum _{i\in N}\alpha _{i}. \end{aligned}$$

We take $||A||=0$ when no game has been played.

The (broadcasting) **problem** is a pair (*A*, *E*), where $A\in {\mathcal { A}}_{n\times n}$ is a matrix defined as above and $E\in \mathbb {R}_{+}$ is an endowment to be allocated among teams in *N*, based on the audience matrix.^5^ The family of all the problems is denoted by ${\mathcal {P}}$. Let ${\mathcal {P}}^{c}$ denote the subset of ${\mathcal {P}}$ encompassing the problems corresponding to fully completed seasons. Namely, $\left( A,E\right) \in {\mathcal {P}}^{c}$ if and only if $a_{ij}\ne \varnothing$, for each pair $i,j\in N$, with $i\ne j$.^6^

### Some illustrative examples

In this section, we discuss in an informal way the main ideas of the paper.

First, we try to clarify with a real-life example (coming from the Spanish professional football league) the broadcasting revenue sharing problem we are considering and the theoretical model associated with it.

Second, we discuss two approaches to solve the problem, when leagues are cancelled. In the first approach, we only use the audiences of the games that were played to solve the problem. In the second approach, we “estimate” first the audiences of the cancelled games. Then, we use the audiences of the games played and the “estimated” audiences of the cancelled games to solve the problem.

Third, we discuss the two rules we consider (equal-split and concede-and-divide) to solve the problem (in each of the two above-mentioned approaches).

The Spanish professional football league is formed by two leagues: “La Liga Santander” (dubbed first division) and “La Liga Smartbank” (dubbed second division). The Spanish law (as it appears in the Official Bulletin of the Spanish State on May 1, 2015) states that the broadcasting rights of both leagues must be jointly sold and that $90\%$ of the total raised revenue must be assigned to the teams playing in the first division. Furthermore, $\frac{1}{6}$th of such an amount is supposed to be divided according to the audiences teams generate (the remainder $\frac{5}{6}$th is divided according to other criteria we shall ignore here). We explain next the procedure to do so, which involves several stages.

Stage 1. Before the beginning of the season, the Spanish professional football league agrees to sell the broadcasting rights to some companies (after a bargaining process between the representatives of the Spanish professional football league and the companies). Let $E^{0}$ be the total amount paid by the companies.

Stage 2. The season takes place. Let *A* be the corresponding matrix of audiences.

Stage 3. Once the season has finished, the Spanish law specifies that $E= \frac{1}{6}\frac{9}{10} E^{0}$ will be allocated among teams in the first division, based on the audiences they generated.

We study Stage 3. Namely, how to divide an amount *E*, among the teams involved, according to the matrix of audiences *A*. Other relevant aspects of the problem, such as how $E^{0}$ is determined (Stage 1), or how the games are scheduled (Stage 2), or the part of the revenues that is assigned to the audiences, are not considered in this paper.

In the theoretical model of the benchmark broadcasting problem (with no cancellations) that we studied in earlier work, we made a simplifying assumption. Instead of dividing the revenue from the sale of broadcasting rights, we divided the total audience of the season $\left( \left| \left| A\right| \right| =\sum _{i,j\in N}a_{ij}\right) .$ Then, the theoretical model only considers matrix *A* as the input of the problem. In our applications to the case of Spain (for instance in Bergantiños and Moreno-Ternero, 2020a) we consider the corresponding scale factor ($\frac{\frac{1}{6}\frac{9}{10} E^{0} }{\left| \left| A\right| \right| }$) to the allocation proposed by the rules we suggest.

Now, suppose that the season is partially cancelled. What happens then to the three stages mentioned above?

Stage 1. Remains the same.

Stage 2. Some games have been played but some others have been cancelled. Furthermore, the sale of broadcasting rights is renegotiated to account for the new situation. Consequently, assume that the available endowment is $\hat{E}^{0}<E^{0}$. In this paper, we do not study the bargaining process leading from $E^{0}$ to $\hat{E}^{0}$. Instead, we simply take $\hat{E}^{0}$ as an input of our problem.

Stage 3. We apply the Spanish law with $\hat{E}^{0}$, instead of $E^{0}$. We also take into account that, now, some games have been cancelled.

Thus, in the theoretical model of the broadcasting problem with cancellations we have two inputs: *A*, the audience matrix (in which some entries could be empty) and *E*, the amount to divide among the teams (i.e., $E=\frac{1}{6}\frac{9}{10} \hat{E}^{0}$).

If we would not have made the simplifying assumption in the benchmark problem, we would also have two inputs: *A*, the audience matrix (in which all entries are non empty) and *E*, the amount to divide among the teams ($E= \frac{1}{6}\frac{9}{10}\hat{E}^{0}$). Notice that the qualitative difference between both problems (with and without cancellations) is in the matrix *A*. In the case of no cancellations, all entries are non empty, whereas in the case of cancellations some entries could be empty. Thus, a relevant issue is how to manage the empty entries of the matrix.

In the following numerical example, we introduce the main ideas of this paper, without entering into technical details (which will be addressed later in the paper).

#### Example 1

Let $\left( A,E\right) \in \mathcal {P}$ be such that $N=\left\{ 1,2,3,4\right\} ,$ $E=100,$ and

$$\begin{aligned} A=\left( \begin{array}{llll} \varnothing &{}\quad \varnothing &{}\quad 10 &{}\quad 10 \\ 10 &{}\quad \varnothing &{}\quad 1 &{}\quad 1 \\ 10 &{}\quad 1 &{}\quad \varnothing &{}\quad \varnothing \\ 10 &{}\quad 1 &{}\quad 1 &{}\quad \varnothing \end{array} \right) . \end{aligned}$$

In this case, we are considering a (double round-robin) league with four teams where the last round of the tournament (games 1–2 and 3–4) was cancelled. Furthermore, one of the teams in the league (team 1) is stronger (in terms of audiences) than the other three (normal) teams that are symmetric. The goal is to divide the generated revenue (100) among the teams using the information from matrix *A*.

In the case without cancellations, one of the rules considered (which we will introduce formally later) divides the revenue proportionally to the audiences of the teams. It seems reasonable to apply the same idea in this example. If so, we have the following:

$$\begin{aligned} \begin{array}{ccccc} &{} \text {Team 1} &{} \text {Team 2} &{} \text {Team 3} &{} \text {Team 4} \\ \text {Audiences} &{} 50 &{} 14 &{} 23 &{} 23 \\ \text {Allocation} &{} 45.5 &{} 12.7 &{} 20.9 &{} 20.9\\ \end{array} \end{aligned}$$

Notice that the previous approach is the same as if we assign a 0 audience to the cancelled games and we compute the rule in the induced problem without cancellations.

One might wonder whether assigning a 0 audience to all cancelled games is the best option. Another reasonable option, for instance, would be to treat all cancelled games equally. But this could be “unfair” for some teams. To wit, in this example, when team 1 plays, the audience is 10 but when team 1 does not play the audience is 1. If we would assign an audience to the cancelled games, then it seems more reasonable to assign an audience of 10 to the cancelled game between teams 1 and 2 and an audience of 1 to the cancelled game between teams 3 and 4. Suppose now that we divide the revenue proportionally to the new assigned audiences. Then, we have the following:

$$\begin{aligned} \begin{array}{ccccc} &{} \text {Team 1} &{} \text {Team 2} &{} \text {Team 3} &{} \text {Team 4} \\ \text {Assigned audiences} &{} 60 &{} 24 &{} 24 &{} 24 \\ \text {Allocation} &{} 45.5 &{} 18.2 &{} 18.2 &{} 18.2 \end{array} \end{aligned}$$

In this particular numerical example, we believe that the allocation provided by the second approach is better than the one provided by the first approach. Given the configuration of this league, we could argue that team 2 is unlucky because it losses the audience from a game against the strongest team, whereas teams 3 and 4 simply lost the audience of a game against a normal team. Thus, it seems fair to treat teams 2, 3 and 4 equally, instead of rendering them contingent on the lottery on how tournament rounds are designed.

The above example illustrates two possible ways to solve broadcasting problems for cancelled seasons. First, to allocate the revenue using only the real audiences from the non-cancelled games (or, equivalently, to assign a 0 audience to the cancelled games). Second, “to estimate” the audiences of the cancelled games and to allocate the revenue using both the real audiences from the non-cancelled games and the “estimated” audiences of the cancelled games.

Now, the question that arises in the second approach described above is how “to estimate” the audiences of the cancelled games. The answer to this question is not unequivocal. In this paper, we propose a plausible option, but some other reasonable ones are also plausible (and we discuss some of them in the final remarks section). The one we study in this paper, formally defined later via the so-called *leg operator*, is motivated by the categories of viewers we considered in Bergantiños and Moreno-Ternero (2020a).^7^ More precisely, we argued therein that viewers of each game can essentially be divided in two categories: those watching the game because they are fans of one of the teams playing and those watching the game because they think that the specific combination of teams renders the game interesting. We refer to them as hard-core (team) fans and neutral (football) fans, respectively.

Suppose that the game between *i* and *j* at *i*’s stadium has been cancelled. We then try “to estimate” $$ a_{ij}.$$ According to the above-mentioned partition of viewers, $$ a_{ij}=h_{i}+h_{j}+n_{ij}$$, where $$h_{i}$$ denotes the hard-core fans of team *i*, $$h_{j}$$ the hard-core fans of team *j*,  and $$n_{ij}$$ the neutral fans (for this game). We can also compute $$a_{ji}$$ similarly. We acknowledge that, in practice, there are relevant factors that might cause $$a_{ij}\ne a_{ji}$$. For instance, the broadcasting time window (it might well be the case that, in the first leg, the game was scheduled in prime time whereas in the second leg it was not), the performance of the teams (it might well be the case that team *i* is doing very well in the first leg but not in the second leg) and so on. In any case, we argue that it is reasonable “to estimate” $$a_{ij}$$ as $$a_{ji}$$, provided such a game was not cancelled too. That is, we take as a proxy of the audience of a game between two teams, the audience of the game between the same teams in the first leg of the tournament.

We finish this discussion by comparing the two rules we shall use to share the revenue. Both rules could be applied in the two ways described above (using only audiences from non-cancelled games or also “estimating” audiences of cancelled games, as outlined previously). As we have explained above, viewers of each game can be partitioned in two categories: hard-core fans and neutral fans. In Bergantiños and Moreno-Ternero (2020a), we argue that the revenue generated by hard-core fans should be allocated to the corresponding team, whereas the revenue generated by neutral fans should be divided equally between both teams. The *equal-split* rule and *concede-and-divide* are two extreme rules from the point of view of treating fans. The * equal-split* rule assumes that only neutral fans exist, whereas * concede-and-divide* assumes that there are as many hard-core fans as possible (compatible with the audiences). We now apply those ideas to an example where the differences between both rules are rather extreme.

#### Example 2

Let $\left( A,E\right) \in \mathcal {P}$ be such that $N=\left\{ 1,2,3,4\right\} ,$ $E=100,$ and

$$\begin{aligned} A=\left( \begin{array}{llll} \varnothing &{}\quad 8 &{}\quad 10 &{}\quad 9 \\ 10 &{}\quad \varnothing &{}\quad \varnothing &{}\quad \varnothing \\ 12 &{}\quad \varnothing &{}\quad \varnothing &{}\quad \varnothing \\ 11 &{}\quad \varnothing &{}\quad \varnothing &{}\quad \varnothing \end{array} \right) . \end{aligned}$$

In this case, we are considering a league with four teams where only the games involving team 1 have been played.

Assume that only neutral fans exist. Then, we should divide the revenue proportionally to the audience of the teams. Thus,

$$\begin{aligned} \begin{array}{ccccc} &{} \text {Team 1} &{} \text {Team 2} &{} \text {Team 3} &{} \text {Team 4} \\ \text {Audiences} &{} 60 &{} 18 &{} 22 &{} 20 \\ \text {Allocation} &{} 50 &{} 15 &{} 18.3 &{} 16.7 \end{array} \end{aligned}$$

Assume now that there are as many hard-core fans as possible (compatible with the audiences). We then assume that team 1 has hard-core fans whereas the other teams do not. Notice that this explanation is compatible with the data and the assumption that no neutral fan exists. Thus, team 1 receives 100 and the rest of the teams receive 0, which is in stark contrast with the previous allocation.

### Benchmark rules

A (sharing) **rule** *R* is a mapping that associates with each problem an allocation indicating the amount each team gets from the endowment. Thus, $R:\mathcal {P}\rightarrow \mathbb {R}^{N}$ is such that, for each $(A,E)\in {\mathcal {P}}$,

$$\begin{aligned} \sum _{i\in N}R_{i}\left( A,E\right) =E. \end{aligned}$$

We also impose from the outset that, if $\left| \left| A\right| \right| =0$, then $R_{i}\left( A,E\right) =\frac{E}{n}$, for each $i\in N$. Furthermore, $R_{i}\left( A,0\right) =0$, for each $i\in N$.

We consider two focal rules for problems in ${\mathcal {P}}^{c}$, which have been introduced in Bergantiños and Moreno-Ternero (2020a). Recall that therein the revenue generated from each viewer is normalized to 1. In other words, the goal is to allocate $\left| \left| A\right| \right|$ among teams. In the context of this paper, the goal is to allocate *E*. Thus, we adapt the definitions of the two rules to our setting.

The *equal-split* rule splits equally the audience of each game $\left( a_{ij}\right)$ among the two teams, thus ignoring the existence of hard-core fans for each team. The total audience assigned to each team is computed as the sum, over all games played by such a team, of the audiences assigned to each game. The amount received by each team is obtained by multiplying the audience assigned to the team by $\frac{E}{\left| \left| A\right| \right| }$, the per capita revenue. Formally,

**Equal-split**, *ES*: for each $(A,E)\in \mathbf { \mathcal {P}}^{c}$, and each $i\in N$,

$$\begin{aligned} ES_{i}\left( A,E\right) =\frac{E}{\left| \left| A\right| \right| }\frac{\alpha _{i}}{2}. \end{aligned}$$

The *equal-split* rule has game-theoretical foundations as, among other things, it coincides with the Shapley value of a suitably associated TU-game to broadcasting problems (e.g. Bergantiños and Moreno-Ternero, 2020a).

The second rule, the so-called *concede-and-divide*, concedes each team its number of fans and divides equally the rest. For each team *i*, we estimate the number of fans of team *i*: $f_{i}$. Given a game with audience $a_{ij},$ *i* receives $f_{i}+\frac{a_{ij}-f_{i}-f_{j}}{2}$ and *j* receives $f_{j}+\frac{a_{ij}-f_{i}-f_{j}}{2}.$ The total audience assigned to each team is computed as the sum over all games played by such a team. Bergantiños and Moreno-Ternero (2020a) prove that this rule could be computed through an easier formula in such a way that we do not need to estimate the number of fans of each team. Finally, the amount received by each team is obtained by multiplying the audience assigned to the team by $\frac{E}{ \left| \left| A\right| \right| },$ the per capita revenue. Formally,

**Concede-and-divide**, *CD*: for each $(A,E)\in {\mathcal {P}}^{c}$, and each $i\in N$,

$$\begin{aligned} CD_{i}\left( A,E\right) =\frac{\left( n-1\right) \alpha _{i}-\left| \left| A\right| \right| }{n-2}\frac{E}{\left| \left| A\right| \right| }. \end{aligned}$$

### Operators

We aim to provide rules that can also address problems with cancelled games. To do so, we extend benchmark rules (such as the ones introduced above) by means of *operators*, associating to each benchmark rule an extended rule resulting from a two-stage procedure: first, the matrix of audiences is modified to replace its empty entries; second, the rule is then used to solve the resulting problem.^8^

Formally, an operator is a mapping $o:{\mathcal {P}}\rightarrow {\mathcal {P}}^{c}$ assigning to each problem $\left( A,E\right) \in {\mathcal {P}}$ a problem $\left( A^{o},E\right) \in {\mathcal {P }}^{c}$ such that $a_{ij}^{o}=a_{ij}$ when $a_{ij}\ne \varnothing .$

We concentrate on the following two operators. First, the one associating to a cancelled game a zero audience. Second, the one associating to a cancelled game the audience of the game in the first leg of the tournament, or zero if such a game was also cancelled. Formally,

**Zero**, *z*: For each pair $i,j\in N$,

$$\begin{aligned} a_{ij}^{z}=\left\{ \begin{array}{ll} 0 &{}\hbox { if }a_{ij}=\varnothing \\ a_{ij} &{} \hbox { if } a_{ij}\ne \varnothing . \end{array} \right. \end{aligned}$$

**Leg**, $\ell$: For each pair $i,j\in N$,

$$\begin{aligned} a_{ij}^{\ell }=\left\{ \begin{array}{ll} a_{ji} &{}\hbox { if }a_{ij}=\varnothing \hbox { and }a_{ji}\ne \varnothing \\ 0 &{}\hbox { if }a_{ij}=\varnothing \hbox { and }a_{ji}=\varnothing \\ a_{ij} &{}\hbox { if }a_{ij}\ne \varnothing . \end{array} \right. \end{aligned}$$

### Extended rules

For each operator *o*, and each benchmark rule *R* on ${\mathcal {P}} ^{c}$, we can define an extended rule $R^{o}$ on ${\mathcal {P}}$ in the obvious way. Namely, for each $(A,E)\in {\mathcal {P}}$,

$$\begin{aligned} R^{o}\left( A,E\right) =R(A^{o},E). \end{aligned}$$

We sometimes refer to $R^{o}$ as the image of the benchmark rule *R* via the operator *o*. Some instances are just the images of the two benchmark rules introduced above, via the two operators also defined above: the * zero-extended equal-split rule* ($ES^{z}$), the *zero-extended concede-and-divide* ($CD^{z}$), the *leg-extended equal-split rule* ($ES^{\ell }$), and the *leg-extended concede-and-divide* ($CD^{\ell }$).

### Axioms

We now consider several axioms of (extended) rules. First, the axiom that says that if a team has a zero audience in all its non-cancelled games, then such a team gets no revenue.^9^ Formally,

**Null team on non-cancelled games **$\left( NTN\right)$: For each $\left( A,E\right) \in \mathcal {P}$ with $\left| \left| A\right| \right| >0$ and each $i\in N$, such that for each $j\in N\backslash \left\{ i\right\}$, $$a_{ij}\in \left\{ 0,\varnothing \right\} \ $$and $a_{ji}\in \left\{ 0,\varnothing \right\} ,$

$$\begin{aligned} R_{i}\left( A,E\right) =0. \end{aligned}$$

The next axiom formalizes a sort of dual principle as it says that if only the games played by one team have positive audience, then such an * essential* team should receive all the endowment. Formally,

**Essential team on non-cancelled games **$\left( ETN\right)$: For each $\left( A,E\right) \in \mathcal {P}$, and each $i\in N$ such that $a_{jk}\in \left\{ 0,\varnothing \right\}$ for each pair $\left\{ j,k\right\} \subset N\backslash \left\{ i\right\} ,$

$$\begin{aligned} R_{i}\left( A,E\right) =E. \end{aligned}$$

We now turn to monotonicity axioms, which are natural in resource allocation.^10^ To motivate them, we consider first the following example.

#### Example 3

Let $$\left( A,E\right) ,\left( A^{\prime },E\right) \in \mathcal {P}$$ be such that $$N=\left\{ 1,2,3,4\right\} ,$$ $$ E=100,$$ and

$$\begin{aligned} {A}=\left( \begin{array}{llll} \varnothing &{}\quad 230 &{}\quad \varnothing &{}\quad 200 \\ \varnothing &{}\quad \varnothing &{}\quad 210 &{}\quad 200 \\ 220 &{}\quad \varnothing &{}\quad \varnothing &{}\quad 200 \\ 200 &{}\quad 200 &{}\quad 200 &{}\quad \varnothing \end{array} \right) \text { and }{A}^{\prime }=\left( \begin{array}{llll} \varnothing &{}\quad 230 &{}\quad 3 &{}\quad 200 \\ \varnothing &{}\quad \varnothing &{}\quad 210 &{}\quad 200 \\ 220 &{}\quad \varnothing &{}\quad \varnothing &{}\quad 200 \\ 200 &{}\quad 200 &{}\quad 200 &{}\quad \varnothing \end{array} \right) \end{aligned}$$

In the latter case, teams 1 and 3 have played one more game than in the former case, with an audience $a_{13}^{\prime }=3.$ How should the allocation of those teams change from one case to the other? Several reasonable answers are possible. For instance,

1. As the *total* audience of team 1 increased, then the allocation to team 1 should not decrease.
2. As the *relative* audience (per game) of team 1 decreased, then the allocation to team 1 should not increase.

Prompted by the above discussion, we introduce a monotonicity property that, depending on a vector of *baseline* audiences, suggests several possible scenarios.^11^ More precisely, *baseline monotonicity* compares the allocation in a problem $\left( A,E\right) \in {\mathcal {P}}$ with the allocation in the resulting problem $\left( A^{\prime },E\right) \in {\mathcal {P}}$ , arising after just modifying the audience of a single game (the (*i*, *j*) entry). If such a game was played in both cases (i.e., $\varnothing \ne a_{ij}\le a_{ij}^{\prime }$) then, in the one with larger audience ($A^{\prime }$), the involved teams (*i*, *j*) cannot receive less, whereas the rest of the teams cannot receive more. If the game was not played in one of the cases (i.e., $a_{ij}=\varnothing \ne a_{ij}^{\prime }$), then we apply the same idea, but now considering the audience assigned by the given operator to the non-played game $\left( a_{ij}^{o}\right)$. Formally,

**O-baseline monotonicity** ($BM^{o}$): Let *o* be an operator and $\left( A,E\right) ,\left( A^{\prime },E\right) \in {\mathcal {P}}$ for which there exist $i,j\in N$ such that $a_{ij}^{\prime }\ne \varnothing$ and $a_{kl}^{\prime }=a_{kl}$ for each $\left( k,l\right) \ne \left( i,j\right) .$ Then, two conditions hold:

1. For each $$k\in \left\{ i,j\right\} ,$$
  $$\begin{aligned} R_{k}\left( A^{\prime },E\right)\ge & {} R_{k}\left( A,E\right) \text { when } a_{ij}^{\prime }\ge a_{ij}^{o}, \\ R_{k}\left( A^{\prime },E\right)\le & {} R_{k}\left( A,E\right) \text { when } a_{ij}^{\prime }\le a_{ij}^{o}. \end{aligned}$$
2. For each $$k\in N\backslash \left\{ i,j\right\} $$,
  $$\begin{aligned} R_{k}\left( A^{\prime },E\right)\le & {} R_{k}\left( A,E\right) \text { when } a_{ij}^{\prime }\ge a_{ij}^{o}, \\ R_{k}\left( A^{\prime },E\right)\ge & {} R_{k}\left( A,E\right) \text { when } a_{ij}^{\prime }\le a_{ij}^{o}. \end{aligned}$$

If a rule satisfies $BM^{z}$, then we say that the rule satisfies * zero baseline monotonicity*. Note that if a rule satisfies $BM^{z}$ then to play a game with zero audience could not be worse than not playing such a game. If a rule satisfies $BM^{\ell }$, then we say that the rule satisfies *leg baseline monotonicity*.^12^

Bergantiños and Moreno-Ternero (2022b) introduce several monotonicity axioms in the benchmark broadcasting problem. Two of them are closely related with *baseline monotonicity*. *Weak team monotonicity* says that for each pair *A*,  $$A^{\prime }$$ and $$i\in N$$ such that $$ a_{ij}\le a_{ij}^{\prime }$$ for all $$j\in N\backslash \left\{ i\right\} ,$$ $$ a_{ji}\le a_{ji}^{\prime }$$ for all $$j\in N\backslash \left\{ i\right\} ,$$ and $$a_{jk}=a_{jk}^{\prime }$$ for all $$j,k\in N\backslash \left\{ i\right\} , $$ then $$R_{i}\left( A^{\prime }\right) \ge R_{i}\left( A\right) .$$ As $$ a_{ij}^{o}=a_{ij}$$ when $$a_{ij}\ne \varnothing ,$$ condition 1 of * o-baseline monotonicity* is an extension of *weak team monotonicity*. *Reciprocal monotonicity* says that for each pair *A*,  $$A^{\prime }$$ and $$i\in N$$ such that $$a_{ij}=a_{ij}^{\prime }$$ for all $$j\in N\backslash \left\{ i\right\} ,$$ $$a_{ji}=a_{ji}^{\prime }$$ for all $$j\in N\backslash \left\{ i\right\} ,$$ and $$a_{jk}\le a_{jk}^{\prime }$$ for all $$j,k\in N\backslash \left\{ i\right\} ,$$ then $$R_{i}\left( A^{\prime }\right) \le R_{i}\left( A\right) .$$ As $$a_{ij}^{o}=a_{ij}$$ when $$a_{ij}\ne \varnothing $$ , condition 2 of *o-baseline monotonicity* is an extension of * reciprocal monotonicity*.

We now move to consider axioms preventing manipulations via reallocations.^13^

Suppose first two tournaments such that the aggregate audience of a given team, as well as the aggregate audience of the rest of the games, coincide. Then, such a given team should receive the same in both tournaments. That is, all viewers of each team are equally important, independently of the team’s game they have viewed. Namely,

**Reallocation proofness** $\left( RP\right)$: Let $\left( A,E\right) ,\left( A^{\prime },E\right) \in {\mathcal {P}}$ and $i\in N$ be such that $\alpha _{i}(A)=\alpha _{i}(A^{\prime })$ and $\left| \left| A\right| \right| =\left| \left| A^{\prime }\right| \right|$. Then,

$$\begin{aligned} R_{i}\left( A,E\right) =R_{i}\left( A^{\prime },E\right) . \end{aligned}$$

The following example captures a possibly disturbing feature with the previous axiom.

#### Example 4

Let $A^{1}$, $$A^{2}\ $$and $A^{3}$ be such that $N=\left\{ 1,2,3,4\right\} ,$ $E=100,$ and

$$\begin{aligned} A^{1}=\left( \begin{array}{llll} \varnothing &{}\quad 100 &{}\quad 60 &{}\quad 50 \\ \varnothing &{}\quad \varnothing &{}\quad 30 &{}\quad 50 \\ 60 &{}\quad 30 &{}\quad \varnothing &{}\quad 50 \\ 50 &{}\quad 50 &{}\quad 50 &{}\quad \varnothing \end{array} \right) , A^{2}=\left( \begin{array}{llll} \varnothing &{}\quad 20 &{}\quad 140 &{}\quad 50 \\ \varnothing &{}\quad \varnothing &{}\quad 30 &{}\quad 50 \\ 60 &{}\quad 30 &{}\quad \varnothing &{}\quad 50 \\ 50 &{}\quad 50 &{}\quad 50 &{}\quad \varnothing \end{array} \right) ,\text { and }A^{3}=\left( \begin{array}{llll} \varnothing &{}\quad 100 &{}\quad 30 &{}\quad 50 \\ \varnothing &{}\quad \varnothing &{}\quad 30 &{}\quad 50 \\ 90 &{}\quad 30 &{}\quad \varnothing &{}\quad 50 \\ 50 &{}\quad 50 &{}\quad 50 &{}\quad \varnothing \end{array} \right) . \end{aligned}$$

The audience of game $\left( 1,2\right)$ is 100 in $A^{1}$ and $A^{3}$, and 20 in $A^{2}$. But a rule satisfying *reallocation proofness* should ignore these numbers (and take into account only the total audience of team 1). Thus, if the rule is defined through an operator *o*,  such an operator cannot depend on the audience of game $\left( 1,2\right)$. But it might be reasonable to do otherwise, therefore distinguishing between $A^{2}$ and the other two problems.

Prompted by the previous example, we weaken *reallocation proofness* by claiming that the allocation to team *i* should be independent of reallocations of audiences but only when no game has been cancelled (namely, on ${\mathcal {P}}^{c})$.

**Weak reallocation proofness** $\left( WRP\right)$: Let $\left( A,E\right) ,\left( A^{\prime },E\right) \in {\mathcal {P}}^{c}$ and $i\in N$ be such that $\alpha _{i}(A)=\alpha _{i}(A^{\prime })$ and $\left| \left| A\right| \right| =\left| \left| A^{\prime }\right| \right|$. Then,

$$\begin{aligned} R_{i}\left( A,E\right) =R_{i}\left( A^{\prime },E\right) . \end{aligned}$$

The last axioms we consider refer to leagues divided into conferences.^14^ We assume that only games among teams in the same conference have a positive audience. An alternative interpretation is that each conference can be seen as a different tournament. Then, instead of solving the whole tournament, we can solve each conference tournament separately, under the assumption that the endowment is divided among the conference tournaments proportionally to their estimated audiences, computed via operator *o*. We consider two axioms, depending on how we define the conference tournament. The following example illustrates this situation.

#### Example 5

Let $\left( A,E\right) \in {\mathcal {P}}$ be such that $N=\left\{ 1,2,3,4,5\right\}$, $E=100$ and

$$\begin{aligned} A=\left( \begin{array}{lllll} \varnothing &{}\quad 100 &{}\quad \varnothing &{}\quad \varnothing &{}\quad \varnothing \\ \varnothing &{}\quad \varnothing &{}\quad \varnothing &{}\quad \varnothing &{}\quad \varnothing \\ \varnothing &{}\quad \varnothing &{}\quad \varnothing &{}\quad 49 &{}\quad \varnothing \\ \varnothing &{}\quad \varnothing &{}\quad 49 &{}\quad \varnothing &{}\quad 1 \\ 0 &{}\quad 0 &{}\quad \varnothing &{}\quad 1 &{}\quad \varnothing \end{array} \right) \end{aligned}$$

In this case, there are two conferences. One with teams 1 and 2 and the other with teams 3, 4, and 5. How do we define the tournaments associated with a conference? We consider two possible options.

1. Only teams from the conference participate in the conference. Namely, $\left\{ 1,2\right\}$ and $\left\{ 3,4,5\right\} .$ Thus, the associated audience matrix is given by
  $$\begin{aligned} \left\{ 1,2\right\} \rightarrow \left( \begin{array}{ll} \varnothing &{}\quad 100 \\ \varnothing &{}\quad \varnothing \end{array} \right) \text { and }\left\{ 3,4,5\right\} \rightarrow \left( \begin{array}{lll} \varnothing &{}\quad 49 &{}\quad \varnothing \\ 49 &{}\quad \varnothing &{}\quad 1 \\ \varnothing &{}\quad 1 &{}\quad \varnothing \end{array} \right) . \end{aligned}$$
2. All teams from the tournament participate in the conference but we only consider the audiences among the teams belonging to the conference. Thus, the associated audience matrix is given by
  $$\begin{aligned} \left\{ 1,2\right\} \rightarrow \left( \begin{array}{lllll} \varnothing &{}\quad 100 &{}\quad \varnothing &{}\quad \varnothing &{}\quad \varnothing \\ \varnothing &{}\quad \varnothing &{}\quad \varnothing &{}\quad \varnothing &{}\quad \varnothing \\ \varnothing &{}\quad \varnothing &{}\quad \varnothing &{}\quad \varnothing &{}\quad \varnothing \\ \varnothing &{}\quad \varnothing &{}\quad \varnothing &{}\quad \varnothing &{}\quad \varnothing \\ \varnothing &{}\quad \varnothing &{}\quad \varnothing &{}\quad \varnothing &{}\quad \varnothing \end{array} \right) \text { and }\left\{ 3,4,5\right\} \rightarrow \left( \begin{array}{lllll} \varnothing &{}\quad \varnothing &{}\quad \varnothing &{}\quad \varnothing &{}\quad \varnothing \\ \varnothing &{}\quad \varnothing &{}\quad \varnothing &{}\quad \varnothing &{}\quad \varnothing \\ \varnothing &{}\quad \varnothing &{}\quad \varnothing &{}\quad 49 &{}\quad \varnothing \\ \varnothing &{}\quad \varnothing &{}\quad 49 &{}\quad \varnothing &{}\quad 1 \\ \varnothing &{}\quad \varnothing &{}\quad \varnothing &{}\quad 1 &{}\quad \varnothing \end{array} \right) . \end{aligned}$$

Formally, for each $\left( A,E\right) \in {\mathcal {P}}$, and each $S\subset N$, we consider two ways of modeling the tournament induced by *A* among teams in *S*:

1. We denote by $$A^{S}\in {\mathcal {A}}_{\left| S\right| \times \left| S\right| }$$ the matrix obtained from *A* by considering that the set of teams is *S* and the audiences are given by *A*. Namely, $$ a_{ij}^{S}=a_{ij}$$ for all $$i,j\in S.$$
2. We denote by $A^{S,\varnothing }\in {\mathcal {A}}_{n\times n}$ the matrix obtained from *A* by assuming that only the games between teams within *S* have been played. Namely,
  $$\begin{aligned} a_{ij}^{S,\varnothing }=\left\{ \begin{array}{ll} a_{ij} &{}\hbox { if }i,j\in S \\ \varnothing &{}\hbox { otherwise}. \end{array} \right. \end{aligned}$$

Notice that in $A^{S}$ the set of teams is *S* whereas in $A^{S,\varnothing }$ the set of teams is *N*.

Before stating the axioms formally, let us illustrate them in Example 5. We have to divide first $E=100$ among the conferences, and then relate the allocation to each team in the initial problem and the associated conference problem.

We first divide *E* among the conferences proportionally to the audiences given by the operator. With the zero operator, the total audience of conference $\left\{ 1,2\right\}$ is 100 and the total audience of conference $\left\{ 3,4,5\right\}$ is also 100. Thus, each conference receives 50 (i.e., *E*/2). With the leg operator, the total audience of conference $\left\{ 1,2\right\}$ is 200 and the total audience of conference $\left\{ 3,4,5\right\}$ is 100. Thus, conference $\left\{ 1,2\right\}$ receives 66.6 (i.e., 2*E*/3) and conference $\left\{ 3,4,5\right\}$ receives 33.3.

We now decide how to relate the allocation of each agent. We distinguish between cases 1 and 2.

1. Case 1 and team 1. As team 1 participates in the conference problem given by $\left\{ 1,2\right\}$ but not by $\left\{ 3,4,5\right\} ,$ we say that the allocation to team 1 in the original problem should be the same as the allocation to team 1 in the problem associated to conference $\left\{ 1,2\right\}$.
2. Case 2 and team 1. As team 1 participates in both conference problems, we say that the allocation to team 1 in the original problem should be the sum of the allocation to team 1 in the problems associated to conferences $\left\{ 1,2\right\}$ and $\left\{ 3,4,5\right\} .$

We now formalize the previous ideas beyond the example. For each $\left( A,E\right) \in {\mathcal {P}}$ and each $S\subset N$, we denote by $\left| \left| A\left( S\right) \right| \right|$ the aggregate audience of all games played among teams within *S*. Namely,

$$\begin{aligned} \left| \left| A\left( S\right) \right| \right| =\sum _{i,j\in S,a_{ij}\ne \varnothing }a_{ij}. \end{aligned}$$

We say that $$\left\{ N_{1},...,N_{p}\right\} $$ is a partition of *N* if $$ N=\bigcup \limits _{k=1}^{p}N_{k}$$, $$N_{k}\cap N_{k^{\prime }}=\varnothing $$ for each pair $$k\ne k^{\prime }$$, and $$N_{k}\ne \varnothing $$ for each $$ k=1,...,p.$$

We are now ready to state the two axioms formally.

**O-single-conference** $\left( SC^{o}\right)$: Let $\left( A,E\right) \in {\mathcal {P}},$ $\left\{ N_{1},...,N_{p}\right\}$ a partition of *N* such that if $a_{ij}>0$ with $i\in N_{i^{\prime }}$ and $j\in N_{j^{\prime }}$ then $i^{\prime }=j^{\prime }$. For each $i\in N_{i^{\prime }},$

$$\begin{aligned} R_{i}\left( A,E\right) =R_{i}\left( A^{N_{i^{\prime }}},\frac{\left| \left| A^{o}\left( N_{i^{\prime }}\right) \right| \right| }{ \sum \limits _{k=1}^{p}\left| \left| A^{o}\left( N_{k}\right) \right| \right| }E\right) . \end{aligned}$$

**O-multi-conference** $\left( MC^{o}\right)$: Let $\left( A,E\right) \in {\mathcal {P}},$ $\left\{ N_{1},...,N_{p}\right\}$ a partition of *N* such that if $a_{ij}>0$ with $i\in N_{i^{\prime }}$ and $j\in N_{j^{\prime }}$ then $i^{\prime }=j^{\prime }$. For each $i\in N,$

$$\begin{aligned} R_{i}\left( A,E\right) =\sum _{k=1}^{p}R_{i}\left( A^{N_{k},\varnothing }, \frac{\left| \left| A^{o}\left( N_{k}\right) \right| \right| }{\sum \limits _{k=1}^{p}\left| \left| A^{o}\left( N_{k}\right) \right| \right| }E\right) . \end{aligned}$$

For the *zero operator* and *leg operator*, we shall denote the previous axioms as $SC^{z}$, $MC^{z}$, $SC^{\ell }$, and $MC^{\ell }$, respectively. Note that the condition of *zero-single-conference* can be rewritten only in terms of *A* as

$$\begin{aligned} R_{i}\left( A,E\right) =R_{i}\left( A^{N_{i^{\prime }}},E\frac{\left| \left| A\left( N_{i^{\prime }}\right) \right| \right| }{ \left| \left| A\right| \right| }\right) . \end{aligned}$$

Similarly, for the zero operator, *zero-multi-conference* can be rewritten only in terms of the matrix with the original audiences *A*. Namely, the condition to be satisfied is

$$\begin{aligned} R_{i}\left( A,E\right) =\sum _{k=1}^{p}R_{i}\left( A^{N_{k},\varnothing },E \frac{\left| \left| A\left( N_{k}\right) \right| \right| }{ \left| \left| A\right| \right| }\right) . \end{aligned}$$

## Characterization results

In this section, we present characterization results for the * zero-extended equal-split* rule, *leg-extended equal-split* rule, *zero-extended concede-and-divide*, and *leg-extended concede-and-divide*. The proofs can be found in the Appendix.

Our first result is a characterization of the *extended equal-split* rule, via the *zero operator*.

### Theorem 1

The following statements hold:

$$\left( a\right) $$
A rule satisfies *reallocation proofness* and *zero-single-conference* if and only if it is the zero-extended *equal-split* rule $ES^{z}$.
$$\left( b\right) $$
A rule satisfies *reallocation proofness, null team for non-cancelled games and* *zero-multi-conference* if and only if it is the zero-extended *equal-split* rule $ES^{z}$.

Resorting to *leg-baseline monotonicity*, but weakening * reallocation proofness*, we obtain instead a characterization of the * extended equal-split* rule, via the *leg operator*.

### Theorem 2

The following statements hold:

$$\left( a\right) $$
A rule satisfies *weak reallocation proofness*, *leg-baseline monotonicity, and leg-single-conference* if and only if it is the leg-extended *equal-split* rule $ES^{\ell }$.
$$\left( b\right) $$
A rule satisfies *weak reallocation proofness*, *leg-baseline monotonicity, null team for non-cancelled games and* *leg-multi-conference* if and only if it is the leg-extended * equal-split* rule $ES^{\ell }$.

We now present the counterpart results for the *extended concede-and-divide*, via the *zero* and *leg operator*, respectively.

### Theorem 3

A rule satisfies *reallocation proofness*, * essential team on non-cancelled games*, and *zero-multi-conference* if and only if it is the zero-extended concede-and-divide $CD^{z}$.

In contrast with Theorem 1, the pair made of * zero-multi-conference* and *essential team for non-cancelled games* cannot be replaced by *zero-single-conference* (which $CD^{z}$ does not satisfy).

### Theorem 4

A rule satisfies *weak reallocation proofness*, *essential team on non-cancelled games*, *leg-multi-conference* and *leg-baseline monotonicity* if and only if it is the leg-extended concede-and-divide $CD^{\ell }$.

In contrast with Theorem 2, the pair made of * leg-multi-conference* and *essential team for non-cancelled games* cannot be replaced by *leg-single-conference* (which is not satisfied by $CD^{\ell }$).

**Table 1:** Performance of the rules with respect to the axioms and characterization results

Axioms/rules	ES$$^{z}$$	CD$$ ^{z}$$	ES$$^{\ell }$$	CD$$ ^{\ell }$$
*NTN*				
*ETN*				
$$BM^{z}$$				
$$BM^{\ell }$$				
*RP*				
*WRP*				
$$SC^{z}$$				
$$MC^{z}$$				
$$SC^{\ell }$$				
$$MC^{\ell }$$				

Table 1 summarizes the performance of the rules considered in the paper with respect to the axioms introduced (we avoid the technical, and non-difficult, proofs). The axioms that were used for each of the characterization results stated above are highlighted in each case.

We conclude with characterizations on the restricted domain $\mathcal {P}^{c}$ , i.e., in the case in which no game has been cancelled. Note that, for each $\left( A,E\right) \in \mathcal {P}^{c}$ and each operator *o*,  $A^{o}=A$. Thus, *reallocation proofness* and *weak reallocation proofness * coincide. Besides, the monotonicity and conference axioms do not depend on the operator and we can remove it from their definitions. Moreover, in the definition of the *multi-conference axiom*, we should change $A^{N_{k},\varnothing }$ to $A^{N_{k},0}.$ Likewise, we avoid adding to the names of the axioms “on non-cancelled games”, as it would be redundant in this restricted domain.

### Theorem 5

The following statements hold:

1. A rule defined on $\mathcal {P}^{c}$ satisfies *reallocation proofness* and single*-conference* if and only if it is the * equal-split* rule.
2. A rule defined on $\mathcal {P}^{c}$ satisfies *reallocation proofness*, *multi-conference* and *null team* if and only if it is the *equal-split* rule.
3. A rule defined on $\mathcal {P}^{c}$ satisfies* reallocation proofness*, *multi-conference* and *essential team* if and only if it is* concede-and-divide*.

Theorem 5 provides some characterizations of the *equal-split* rule and *concede-and-divide* in the benchmark case. Bergantiños and Moreno-Ternero (2020a, 2020b, 2022a) also provide several characterizations of the same two rules in such a setting by using, among others, the axioms of *null team* and *essential team*. The axioms of *reallocation proofness*, *single-conference* and *multi-conference* are used in this paper but not in the previous ones.

## Final remarks

We have explored in this paper the allocation of revenues raised from selling broadcasting rights for sports leagues, after some matches have been cancelled. We have provided normative foundations for the extension of two focal rules ( *equal-split* and *concede-and-divide*) via two natural operators: the *zero operator* and the *leg operator*. The former assigns a zero audience to cancelled games. The latter assigns the audience of the corresponding game in the first leg of the tournament to cancelled games. Other operators could also be considered. For instance, the *leg operator* assigns the audience of the corresponding game in the first leg of the tournament when it has not been cancelled (which seems reasonable) and zero when both games have been cancelled (which seems more controversial). Thus, it makes sense to consider alternative definitions of the leg operator for the case where both games have been cancelled. Instances are to associate each cancelled game the audience of the (non-cancelled) game with highest or lowest audience; or the average audience of all the (non-cancelled) games in the tournament. Exploring those operators is left for further research.

Likewise, the two rules we have extended are not only focal but also somewhat extreme in the benchmark setting of complete leagues. Compromises among them have been considered (e.g. Bergantiños and Moreno-Ternero 2021, 2022a, b, c, d, 2023a). It is also left for further research to extend those compromises to the case of cancelled competitions.

We conclude stating that our paper can provide new insights for the literature on the economic design of sporting contests (e.g. Szymanski, 2003), with a particular emphasis on the literature on revenue sharing in contest models. None of those models addresses the issue of cancelled contests, which we explore here.

## Appendix

### Proof of Theorem 1

$\left( a\right)$ It is not difficult to show that $ES^{z}$ satisfies the two axioms in the statement. Conversely, let *R* be a rule satisfying the two axioms. Let $\left( A,E\right) \in {\mathcal {P}}$ be such that $\left| \left| A\right| \right| >0$. Otherwise, the proof is trivial as $R_{i}\left( A,E\right) =\frac{E}{n}$, for each $i\in N$. Let $i\in N.$ Notice that $\left| \left| A\right| \right| -\alpha _{i}\left( A\right)$ is the aggregate audience of all games not played by team *i*. We then define the matrix $A^{*}$ as follows. Let $i^{0},i^{1},i^{2}\in N\backslash \left\{ i\right\} .$ Then,

$$\begin{aligned} a_{jk}^{*}=\left\{ \begin{array}{ll} \frac{\alpha _{i}\left( A\right) }{2} &{}\hbox { if }\left( j,k\right) \in \left\{ \left( i,i^{0}\right) ,\left( i^{0},i\right) \right\} \\ \left| \left| A\right| \right| -\alpha _{i}\left( A\right) &{}\hbox { if }\left( j,k\right) =\left( i^{1},i^{2}\right) \\ \varnothing &{}\hbox { otherwise}. \end{array} \right. \end{aligned}$$

Notice that $\left| \left| A^{*}\right| \right| =\left| \left| A\right| \right| >0$ and $\alpha _{i}\left( A^{*}\right) =\alpha _{i}\left( A\right) .$ Therefore, by* reallocation proofness*, $R_{i}\left( A,E\right) =R_{i}\left( A^{*},E\right) .$

Let $N_{1}=\left\{ i,i^{0}\right\}$ and $N_{2}=N\backslash N_{1}.$ By *zero-single-conference*,

$$\begin{aligned} R_{i}\left( A^{*},E\right)= & {} R_{i}\left( \left( A^{*}\right) ^{N_{1}},\frac{\left| \left| \left( A^{*}\right) ^{z}\left( N_{1}\right) \right| \right| }{\left| \left| \left( A^{*}\right) ^{z}\left( N_{1}\right) \right| \right| +\left| \left| \left( A^{*}\right) ^{z}\left( N_{2}\right) \right| \right| }E\right) \\= & {} R_{i}\left( \left( A^{*}\right) ^{N_{1}},\frac{\alpha _{i}\left( A\right) }{\left| \left| A\right| \right| }E\right) . \end{aligned}$$

As

$$\begin{aligned} R_{i}\left( \left( A^{*}\right) ^{N_{1}},\frac{\alpha _{i}\left( A\right) }{\left| \left| A\right| \right| }E\right) +R_{i^{0}}\left( \left( A^{*}\right) ^{N_{1}},\frac{\alpha _{i}\left( A\right) }{\left| \left| A\right| \right| }E\right) =\frac{ \alpha _{i}\left( A\right) }{\left| \left| A\right| \right| } E, \end{aligned}$$

it follows that there exists $p_{\left( i,i_{0}\right) }\in \mathbb {R}$ such that

$$\begin{aligned} R_{i}\left( \left( A^{*}\right) ^{N_{1}},\frac{\alpha _{i}\left( A\right) }{\left| \left| A\right| \right| }E\right)= & {} p_{\left( i,i_{0}\right) }\frac{\alpha _{i}\left( A\right) }{\left| \left| A\right| \right| }E\text { and } \\ R_{i^{0}}\left( \left( A^{*}\right) ^{N_{1}},\frac{\alpha _{i}\left( A\right) }{\left| \left| A\right| \right| }E\right)= & {} \left( 1-p_{\left( i,i_{0}\right) }\right) \frac{\alpha _{i}\left( A\right) }{\left| \left| A\right| \right| }E. \end{aligned}$$

As $R_{i}\left( A,E\right) =R_{i}\left( \left( A^{*}\right) ^{N_{1}}, \frac{\alpha _{i}\left( A\right) }{\left| \left| A\right| \right| }E\right) =p_{\left( i,i_{0}\right) }\frac{\alpha _{i}\left( A\right) }{\left| \left| A\right| \right| }E$, for each $i^{0}\in N\backslash \left\{ i\right\} ,$ we deduce that $p_{\left( i,i_{0}\right) }$ is indeed independent of $i_{0}$. Thus, we can just refer to it as $p_{i}$.

If $$\alpha _{i}\left( A\right) =0,$$ $$R_{i}\left( A,E\right) =0=ES_{i}\left( A,E\right) .$$

Therefore, assume now that $\alpha _{i}\left( A\right) >0.$ Let $i^{\prime }\in N\backslash \left\{ i\right\}$ and construct the problem $\left( A^{\prime },E\right)$ such that $\alpha _{i^{\prime }}\left( A^{\prime }\right) =\alpha _{i}\left( A\right)$ and $\left| \left| A^{\prime }\right| \right| =\left| \left| A\right| \right| .$

We then construct the matrix $A^{\prime *}$, analogously to how we constructed $A^{*}$ from *A*, but now assigning the former role of *i* to $i^{\prime }$, and the former role of $i^{0}$ to *i*. That is,

$$\begin{aligned} a_{jk}^{\prime *}=\left\{ \begin{array}{ll} \frac{\alpha _{i}\left( A^{\prime }\right) }{2} &{}\hbox { if }\left( j,k\right) \in \left\{ \left( i^{\prime },i\right) ,\left( i,i^{\prime }\right) \right\} \\ \left| \left| A^{\prime }\right| \right| -\alpha _{i}\left( A^{\prime }\right) &{}\hbox { if }\left( j,k\right) =\left( i^{1},i^{2}\right) \\ \varnothing &{}\hbox { otherwise}. \end{array} \right. \end{aligned}$$

Then,

$$\begin{aligned} p_{i}\frac{\alpha _{i}\left( A\right) }{\left| \left| A\right| \right| }E=R_{i}\left( \left( A^{*}\right) ^{N_{1}},\frac{\alpha _{i}\left( A\right) }{\left| \left| A\right| \right| } E\right) =R_{i}\left( \left( A^{\prime *}\right) ^{N_{1}},\frac{\alpha _{i^{\prime }}\left( A^{\prime }\right) }{\left| \left| A^{\prime }\right| \right| }E\right) =\left( 1-p_{i^{\prime }}\right) \frac{ \alpha _{i^{\prime }}\left( A^{\prime }\right) }{\left| \left| A^{\prime }\right| \right| }E, \end{aligned}$$

from where it follows that $p_{i}=1-p_{i^{\prime }}.$

As *i* and $i^{\prime }$ were arbitrary members of *N*, it follows that $p_{j}=\frac{1}{2}$ for all $j\in N$. Hence,

$$\begin{aligned} R_{i}\left( A,E\right) =R_{i}\left( \left( A^{1}\right) ^{N_{1}},\frac{ \alpha _{i}\left( A\right) }{\left| \left| A\right| \right| } E\right) =p_{i}\frac{\alpha _{i}\left( A\right) }{\left| \left| A\right| \right| }E=\frac{1}{2}\frac{\alpha _{i}\left( A\right) }{ \left| \left| A\right| \right| }E=ES_{i}^{z}\left( A,E\right) . \end{aligned}$$

$\left( b\right)$ It is straightforward to prove that $ES^{z}$ also satisfies *zero-multi-conference* and *null team on non-cancelled games*. As for the converse implication, note that * zero-single-conference* is used only once in the previous proof. We explain now how to derive the same conclusion therein with * zero-multi-conference* and *null team on non-cancelled games*.

By *zero-multi-conference*,

$$\begin{aligned} R_{i}\left( A^{*},E\right) =R_{i}\left( \left( A^{*}\right) ^{N_{1},\varnothing },\frac{\alpha _{i}\left( A\right) }{\left| \left| A\right| \right| }E\right) +R_{i}\left( \left( A^{*}\right) ^{N_{2},\varnothing },\frac{\left| \left| A\right| \right| -\alpha _{i}\left( A\right) }{\left| \left| A\right| \right| }E\right) . \end{aligned}$$

By *null team on non-cancelled games*, $R_{i}\left( \left( A^{*}\right) ^{N_{2},\varnothing },\frac{\left| \left| A\right| \right| -\alpha _{i}\left( A\right) }{\left| \left| A\right| \right| }E\right) =0.$ Hence, $R_{i}\left( A^{*},E\right) =R_{i}\left( \left( A^{*}\right) ^{N_{1},\varnothing },\frac{\alpha _{i}\left( A\right) }{\left| \left| A\right| \right| } E\right)$.

By *null team on non-cancelled games*, $R_{j}\left( \left( A^{*}\right) ^{N_{1},\varnothing },\frac{\alpha _{i}\left( A\right) }{\left| \left| A\right| \right| }E\right) =0$ for all $j\in N^{2}.$ Using arguments similar to those used above for $\left( \left( A^{*}\right) ^{N_{1}},\frac{\alpha _{i}\left( A\right) }{\left| \left| A\right| \right| }E\right)$ we can prove that $R_{i}\left( \left( A^{*}\right) ^{N_{1},\varnothing },\frac{\alpha _{i}\left( A\right) }{ \left| \left| A\right| \right| }E\right) =\frac{\alpha _{i}\left( A\right) }{2\left| \left| A\right| \right| } E=ES_{i}^{z}\left( A,E\right) .$ $\square$

### Proof of Theorem 2

$\left( a\right)$ It is straightforward to show that $ES^{\ell }$ satisfies *weak reallocation proofness* and *leg-single-conference*. Regarding *leg-baseline monotonicity*, let $\left( A,E\right) ,\left( A^{\prime },E\right) \in {\mathcal {P}}$ and $i,j\in N$ be as in the definition of this axiom. We consider several cases.

1. $$a_{ij}=\varnothing \ne a_{ji}.$$
  In this case, $a_{ij}^{\ell }=a_{ji}.$ Thus,
  $$\begin{aligned} \alpha _{i}\left( A^{\prime \ell }\right)= & {} \alpha _{i}\left( A^{\ell }\right) +a_{ij}^{\prime }-a_{ji} \\ \alpha _{j}\left( A^{\prime \ell }\right)= & {} \alpha _{j}\left( A^{\ell }\right) +a_{ij}^{\prime }-a_{ji} \\ \alpha _{k}\left( A^{\prime \ell }\right)= & {} \alpha _{k}\left( A^{\ell }\right) \text { for all }k\in N\backslash \left\{ i,j\right\} \text { and } \\ \left| \left| A^{\prime \ell }\right| \right|= & {} \left| \left| A^{\ell }\right| \right| +a_{ij}^{\prime }-a_{ji}. \end{aligned}$$
  We consider two sub-cases.
  - $$a_{ij}^{\prime }\ge a_{ji}.$$
    In this case, $ES_{k}\left( A^{\prime l},E\right) \ge ES_{k}\left( A^{\ell },E\right)$, for each $k\in \left\{ i,j\right\} .$ Besides, $a_{ij}^{\prime }\ge a_{ji}=a_{ij}^{\ell }$ and $ES_{k}\left( A^{\prime l},E\right) \le ES_{k}\left( A^{\ell },E\right)$ for each $k\in N\backslash \left\{ i,j\right\} .$
  - $$a_{ij}^{\prime }\le a_{ji}.$$
    In this case, $ES_{k}\left( A^{\prime l},E\right) \le ES_{k}\left( A^{\ell },E\right)$, for each $k\in \left\{ i,j\right\} .$ Besides, $a_{ij}^{\prime }\le a_{ji}=a_{ij}^{\ell }$ and $ES_{k}\left( A^{\prime l},E\right) \ge ES_{k}\left( A^{\ell },E\right)$ for each $k\in N\backslash \left\{ i,j\right\} .$
2. $$a_{ij}=\varnothing =a_{ji}$$.
  In this case, $a_{ij}^{\ell }=0.$ Thus,
  $$\begin{aligned} \alpha _{i}\left( A^{\prime \ell }\right)= & {} \alpha _{i}\left( A^{\ell }\right) +2a_{ij}^{\prime } \\ \alpha _{j}\left( A^{\prime \ell }\right)= & {} \alpha _{j}\left( A^{\ell }\right) +2a_{ij}^{\prime } \\ \alpha _{k}\left( A^{\prime \ell }\right)= & {} \alpha _{k}\left( A^{\ell }\right) \text { for all }k\in N\backslash \left\{ i,j\right\} \text { and } \\ \left| \left| A^{\prime \ell }\right| \right|= & {} \left| \left| A^{\ell }\right| \right| +2a_{ij}^{\prime }. \end{aligned}$$
  Therefore, $ES_{k}\left( A^{\prime l},E\right) \ge ES_{k}\left( A^{\ell },E\right)$ for each $k\in \left\{ i,j\right\} .$ Besides, $a_{ij}^{\prime }\ge 0=a_{ij}^{\ell }$ and $ES_{k}\left( A^{\prime l},E\right) \le ES_{k}\left( A^{\ell },E\right)$ for each $k\in N\backslash \left\{ i,j\right\} .$
3. $$a_{ij}\ne \varnothing \ne a_{ji}$$.
  In this case, $a_{ij}^{\ell }=a_{ij}.$ Thus,
  $$\begin{aligned} \alpha _{i}\left( A^{\prime \ell }\right)= & {} \alpha _{i}\left( A^{\ell }\right) +a_{ij}^{\prime }-a_{ij} \\ \alpha _{j}\left( A^{\prime \ell }\right)= & {} \alpha _{j}\left( A^{\ell }\right) +a_{ij}^{\prime }-a_{ij} \\ \alpha _{k}\left( A^{\prime \ell }\right)= & {} \alpha _{k}\left( A^{\ell }\right) \text { for all }k\in N\backslash \left\{ i,j\right\} \text { and } \\ \left| \left| A^{\prime \ell }\right| \right|= & {} \left| \left| A^{\ell }\right| \right| +a_{ij}^{\prime }-a_{ij}. \end{aligned}$$
  The rest of the proof is similar to that of Case 1.
4. $$a_{ij}\ne \varnothing $$ and $$a_{ji}=\varnothing .$$
  In this case, $a_{ij}^{\ell }=a_{ij}.$ Thus,
  $$\begin{aligned} \alpha _{i}\left( A^{\prime \ell }\right)= & {} \alpha _{i}\left( A^{\ell }\right) +2\left( a_{ij}^{\prime }-a_{ij}\right) \\ \alpha _{j}\left( A^{\prime \ell }\right)= & {} \alpha _{j}\left( A^{\ell }\right) +2\left( a_{ij}^{\prime }-a_{ij}\right) \\ \alpha _{k}\left( A^{\prime \ell }\right)= & {} \alpha _{k}\left( A^{\ell }\right) \text { for all }k\in N\backslash \left\{ i,j\right\} \text { and } \\ \left| \left| A^{\prime \ell }\right| \right|= & {} \left| \left| A^{\ell }\right| \right| +2\left( a_{ij}^{\prime }-a_{ij}\right) . \end{aligned}$$
  The rest of the proof is similar to that of Case 1.

Conversely, let *R* be a rule satisfying all the properties in the statement. Let $\left( A,E\right) \in {\mathcal {P}}$ be such that $\left| \left| A\right| \right| >0$. Otherwise, the proof is trivial as $R_{i}\left( A,E\right) =\frac{E}{n}$, for each $i\in N$. Let $i\in N.$

Let $m\left( A\right)$ be the number of cancelled games in tournament *A*. We prove that $R\left( A,E\right) =ES^{\ell }\left( A,E\right)$ by induction on $m\left( A\right) .$ Assume first that $m\left( A\right) =0$. Thus, $a_{ij}\ne \varnothing$ for all $i,j\in N$ with $i\ne j.$

Let $$i^{0},i^{1},i^{2}\in N\backslash \left\{ i\right\} .$$ We define $$ A^{*}$$ as follows.

$$\begin{aligned} a_{jk}^{*}=\left\{ \begin{array}{ll} \frac{\alpha _{i}\left( A\right) }{2} &{}\hbox { if }\left( j,k\right) \in \left\{ \left( i,i^{0}\right) ,\left( i^{0},i\right) \right\} \\ \left| \left| A\right| \right| -\alpha _{i}\left( A\right) &{}\hbox { if }\left( j,k\right) =\left( i^{1},i^{2}\right) \\ 0 &{}\hbox { otherwise}. \end{array} \right. \end{aligned}$$

Notice that $\left| \left| A^{*}\right| \right| =\left| \left| A\right| \right| >0$ and $\alpha _{i}\left( A^{*}\right) =\alpha _{i}\left( A\right) .$ Therefore, by *weak reallocation proofness*, $R_{i}\left( A^{*},E\right) =R_{i}\left( A,E\right) .$

Let $N_{1}=\left\{ i,i^{0}\right\}$ and $N_{2}=N\backslash N_{1}.$ As $m\left( A^{*}\right) =0$, we deduce that $A^{*l}=A^{*}.$ Besides, $\left| \left| A\right| \right| =\left| \left| A^{*}\left( N_{1}\right) \right| \right| +\left| \left| A^{*}\left( N_{2}\right) \right| \right|$ and $\left| \left| A^{*}\left( N_{1}\right) \right| \right| =\alpha _{i}\left( A\right) .$ By *leg-single-conference*,

$$\begin{aligned} R_{i}\left( A^{*},E\right) =R_{i}\left( \left( A^{*}\right) ^{N_{1}}, \frac{\alpha _{i}\left( A\right) }{\left| \left| A\right| \right| }E\right) . \end{aligned}$$

Now, using arguments similar to those used in the proof of Theorem 1, we can deduce that

$$\begin{aligned} R_{i}\left( \left( A^{*}\right) ^{N_{1}},\frac{\alpha _{i}\left( A\right) }{\left| \left| A\right| \right| }E\right) =\frac{ \alpha _{i}\left( A\right) }{2\left| \left| A\right| \right| }E=ES_{i}\left( A,E\right) =ES_{i}^{\ell }\left( A,E\right) . \end{aligned}$$

Therefore, $R\left( A,E\right) =ES^{\ell }\left( A,E\right)$ for $m\left( A\right) =0.$ Assume then that $R\left( A,E\right) =ES^{\ell }\left( A,E\right)$ for $m\left( A\right) =p$. Let $\left( A,E\right)$ be such that $m\left( A\right) =p+1.$ Then, there exists a pair $i,j\in N$ such that $a_{ij}=\varnothing .$ We consider two cases.

1. $$a_{ji}\ne \varnothing .$$
  In this case, let $A^{\prime }$ be such that
  $$\begin{aligned} a_{kl}^{\prime }=\left\{ \begin{array}{ll} a_{ji} &{}\hbox { if }\left( k,l\right) =\left( i,j\right) \\ a_{kl} &{}\hbox { otherwise}. \end{array} \right. \end{aligned}$$
  Thus, $m\left( A^{\prime }\right) =p$. By the induction hypothesis,
  $$\begin{aligned} R\left( A^{\prime },E\right) =ES^{\ell }\left( A^{\prime },E\right) =ES\left( A^{\prime l},E\right) =ES\left( A^{\ell },E\right) =ES^{\ell }\left( A,E\right) . \end{aligned}$$
  As $a_{ij}^{\ell }=a_{ji},$ and *R* satisfies *leg-baseline monotonicity*, we deduce that $R_{i}\left( A,E\right) =R_{i}\left( A^{\prime },E\right) .$ Similarly, we can argue that $R_{j}\left( A,E\right) =R_{j}\left( A^{\prime },E\right) .$
  As $a_{ij}^{\ell }=a_{ji}$ and $a_{ij}^{\prime }=a_{ji}$, we deduce that
  $$\begin{aligned} a_{ij}^{\ell }\le a_{ij}^{\prime }\le a_{ij}^{\ell }. \end{aligned}$$
  By *leg-baseline monotonicity*, for each $k\in N\backslash \left\{ i,j\right\}$,
  $$\begin{aligned} R_{k}\left( A^{\prime },E\right) \le R_{k}\left( A,E\right) \le R_{k}\left( A^{\prime },E\right) , \end{aligned}$$
  Thus, $$R\left( A,E\right) =R\left( A^{\prime },E\right) =ES^{\ell }\left( A,E\right) .$$
2. $$a_{ji}=\varnothing .$$
  In this case, let $A^{\prime }$ be such that
  $$\begin{aligned} a_{kl}^{\prime }=\left\{ \begin{array}{ll} 0 &{}\hbox { if }\left( k,l\right) =\left( i,j\right) \\ a_{kl} &{}\hbox { otherwise}. \end{array} \right. \end{aligned}$$
  Replicating the arguments used in Case 1, we can argue that $R\left( A,E\right) =R\left( A^{\prime },E\right) =ES^{\ell }\left( A,E\right) .$

$\left( b\right)$ It is straightforward to prove that $ES^{\ell }$ also satisfies *leg-multi-conference* and *null team on non-cancelled games*. As for the converse implication, note that * leg-single-conference* is used only once in the previous proof. We explain now how to derive the same conclusion therein with * leg-multi-conference* and *null team on non-cancelled games*.

By *leg-multi-conference*,

$$\begin{aligned} R_{i}\left( A^{*},E\right) =R_{i}\left( \left( A^{*}\right) ^{N_{1},\varnothing },\frac{\alpha _{i}\left( A\right) }{\left| \left| A\right| \right| }E\right) +R_{i}\left( \left( A^{*}\right) ^{N_{2},\varnothing },\frac{\left| \left| A\right| \right| -\alpha _{i}\left( A\right) }{\left| \left| A\right| \right| }E\right) . \end{aligned}$$

By *null team on non-cancelled games*, $R_{i}\left( \left( A^{*}\right) ^{N_{2},\varnothing },\frac{\left| \left| A\right| \right| -\alpha _{i}\left( A\right) }{\left| \left| A\right| \right| }E\right) =0.$ Hence, $R_{i}\left( A^{*},E\right) =R_{i}\left( \left( A^{*}\right) ^{N_{1},\varnothing },\frac{\alpha _{i}\left( A\right) }{\left| \left| A\right| \right| } E\right)$.

By *null team on non-cancelled games*, $R_{j}\left( \left( A^{*}\right) ^{N_{1},\varnothing },\frac{\alpha _{i}\left( A\right) }{\left| \left| A\right| \right| }E\right) =0$ for all $j\in N_{2}.$ With similar arguments to those used above for $\left( \left( A^{*}\right) ^{N_{1}},\frac{\alpha _{i}\left( A\right) }{\left| \left| A\right| \right| }E\right)$ we can prove that $R_{i}\left( \left( A^{*}\right) ^{N_{1},\varnothing },\frac{\alpha _{i}\left( A\right) }{ \left| \left| A\right| \right| }E\right) =\frac{\alpha _{i}\left( A\right) }{2\left| \left| A\right| \right| } E=ES_{i}^{\ell }\left( A,E\right) .$ $\square$

### Proof of Theorem 3

It is straightforward to show that $CD^{z}$ satisfies *reallocation proofness* and *essential on non-cancelled games*. Regarding * zero-multi-conference*, let $\left( A,E\right) \in {\mathcal {P}}$ and $\left\{ N_{1},...,N_{p}\right\}$ be as in its definition. Given $i\in N_{i^{\prime }},$

$$\begin{aligned}{} & {} \sum _{k=1}^{p}CD_{i}^{z}\left( A^{N_{k},\varnothing },\frac{\left| \left| A^{z}\left( N_{k}\right) \right| \right| }{ \sum \limits _{k=1}^{p}\left| \left| A^{z}\left( N_{k}\right) \right| \right| }E\right) \\{} & {} \quad =\sum _{k=1}^{p}\frac{\left( n-1\right) \alpha _{i}\left( \left( A^{N_{k},\varnothing }\right) ^{z}\right) -\left| \left| \left( A^{N_{k},\varnothing }\right) ^{z}\right| \right| }{n-2}\frac{\frac{\left| \left| A^{z}\left( N_{k}\right) \right| \right| }{\sum \limits _{k=1}^{p}\left| \left| A^{z}\left( N_{k}\right) \right| \right| }E}{\left| \left| \left( A^{N_{k},\varnothing }\right) ^{z}\right| \right| }. \end{aligned}$$

As

$$\begin{aligned} \alpha _{i}\left( \left( A^{N_{k},\varnothing }\right) ^{z}\right)= & {} \left\{ \begin{array}{ll} \alpha _{i}\left( A^{z}\right) &{}\hbox { if }i\in N_{k} \\ 0 &{} \hbox { otherwise}, \end{array} \right. \\ \left| \left| A^{z}\left( N_{k}\right) \right| \right|= & {} \left| \left| \left( A^{N_{k},\varnothing }\right) ^{z}\right| \right| ,\text { and} \\ \left| \left| A^{z}\right| \right|= & {} \sum \limits _{k=1}^{p}\left| \left| A^{z}\left( N_{k}\right) \right| \right| \end{aligned}$$

it follows that

$$\begin{aligned} \sum _{k=1}^{p}CD_{i}^{z}\left( A^{N_{k},\varnothing },\frac{\left| \left| A^{z}\left( N_{k}\right) \right| \right| }{ \sum \limits _{k=1}^{p}\left| \left| A^{z}\left( N_{k}\right) \right| \right| }E\right)= & {} \frac{\left( n-1\right) \alpha _{i}\left( A^{z}\right) -\left| \left| A^{z}\left( N_{i^{\prime }}\right) \right| \right| }{n-2}\frac{E}{\left| \left| A^{z}\right| \right| }\\{} & {} -\sum _{k\ne i^{\prime }}\frac{\left| \left| A^{z}\left( N_{k}\right) \right| \right| }{n-2}\frac{E}{ \left| \left| A^{z}\right| \right| } \\= & {} \frac{\left( n-1\right) \alpha _{i}\left( A^{z}\right) -\sum \limits _{k=1}^{m}\left| \left| A^{z}\left( N_{k}\right) \right| \right| }{n-2}\frac{E}{\left| \left| A^{z}\right| \right| } \\= & {} \frac{\left( n-1\right) \alpha _{i}\left( A^{z}\right) -\left| \left| A^{z}\right| \right| }{n-2}\frac{E}{\left| \left| A^{z}\right| \right| } \\= & {} CD_{i}\left( A^{z},E\right) =CD_{i}^{z}\left( A,E\right) . \end{aligned}$$

Conversely, let *R* be a rule satisfying all the axioms in the statement. Let $\left( A,E\right) \in {\mathcal {P}}$ be such that $\left| \left| A\right| \right| >0$. Otherwise, the proof is trivial as $R_{i}\left( A,E\right) =\frac{E}{n}$, for each $i\in N$. Let $i\in N.$

Let $A^{*},$ $i^{0},$ $i^{1},$ $i^{2},$ $N_{1}$ and $N_{2}$ be defined as in the proof of Theorem 1. By *reallocation proofness* $R_{i}\left( A,E\right) =R_{i}\left( A^{*},E\right)$. By * zero-multi-conference*,

$$\begin{aligned} R_{i}\left( A^{*},E\right) =R_{i}\left( \left( A^{*}\right) ^{N_{1},\varnothing },\frac{\left| \left| A^{*}\left( N_{1}\right) \right| \right| }{\left| \left| A\right| \right| }E\right) +R_{i}\left( \left( A^{*}\right) ^{N_{2},\varnothing },\frac{\left| \left| A^{*}\left( N_{2}\right) \right| \right| }{\left| \left| A\right| \right| }E\right) . \end{aligned}$$

We first analyze the problem induced by conference $N_{1}.$ We consider two cases.

Case 1.1. $\alpha _{i}\left( A\right) =0.$ Then $\left| \left| A^{*}\left( N_{1}\right) \right| \right| =0$ and hence

$$\begin{aligned} R_{i}\left( \left( A^{*}\right) ^{N_{1},\varnothing },\frac{\left| \left| A^{*}\left( N_{1}\right) \right| \right| }{\left| \left| A\right| \right| }E\right) =0. \end{aligned}$$

Case 1.2. $\alpha _{i}\left( A\right) >0.$ Then $\left| \left| A^{*}\left( N_{1}\right) \right| \right| =\alpha _{i}\left( A\right) >0.$ By *essential team on non-cancelled games*,

$$\begin{aligned} R_{i}\left( \left( A^{*}\right) ^{N_{1},\varnothing },\frac{\left| \left| A^{*}\left( N_{1}\right) \right| \right| }{\left| \left| A\right| \right| }E\right) =\frac{\left| \left| A^{*}\left( N_{1}\right) \right| \right| }{\left| \left| A\right| \right| }E=\frac{\alpha _{i}\left( A\right) }{\left| \left| A\right| \right| }E. \end{aligned}$$

We now analyze the problem induced by conference $N_{2}.$ We also consider two cases.

Case 2.1. $\alpha _{i}\left( A\right) =\left| \left| A\right| \right| .$ Then $\left| \left| A^{*}\left( N_{2}\right) \right| \right| =0$ and hence

$$\begin{aligned} R_{i}\left( \left( A^{*}\right) ^{N_{2},\varnothing },\frac{\left| \left| A^{*}\left( N_{2}\right) \right| \right| }{\left| \left| A\right| \right| }E\right) =0. \end{aligned}$$

Case 2.2. $$\alpha _{i}\left( A\right) <\left| \left| A\right| \right| .$$ Then $$\left| \left| A^{*}\left( N_{2}\right) \right| \right| =\left| \left| A\right| \right| -\alpha _{i}\left( A\right) >0.$$

For each pair $j,k\in N$ and $x\in \mathbb {R}_{+},$ we define $A^{jk,x}$, where

$$\begin{aligned} a_{lm}^{jk,x}=\left\{ \begin{array}{ll} x &{} \hbox { if } \left( l,m\right) =\left( j,k\right) \\ \varnothing &{}\hbox { otherwise}. \end{array} \right. \end{aligned}$$

By *essential team on non-cancelled games*,

$$\begin{aligned} R_{j}\left( A^{jk,x},E\right) =R_{k}\left( A^{jk,x},E\right) =E. \end{aligned}$$

Thus,

1 $$\begin{aligned} \sum _{l\in N\backslash \left\{ j,k\right\} }R_{\ell }\left( A^{jk,x},E\right) =-E. \end{aligned}$$

Let $l\in N.$ Consider $j,k\in N\backslash \left\{ l\right\}$ and $j^{\prime },k^{\prime }\in N\backslash \left\{ l\right\} .$ By * reallocation proofness*,

$$\begin{aligned} R_{\ell }\left( A^{jk,x},E\right) =R_{\ell }\left( A^{j^{\prime }k^{\prime },x},E\right) . \end{aligned}$$

As $R_{\ell }\left( A^{jk,x},E\right)$ does not depend on *j* and *k*, we can define $f\left( l,x,E\right) =$ $R_{\ell }\left( A^{jk,x},E\right)$ for each $l\in N$ and $x,E\in \mathbb {R}$.

Let $j,k\in N$ and $m\in N\backslash \left\{ j,k\right\} .$ By (1),

$$\begin{aligned} -E= & {} \sum _{l\in N\backslash \left\{ j,k,m\right\} }R_{\ell }\left( A^{jk,x},E\right) +R_{m}\left( A^{jk,x},E\right) \text { and} \\ -E= & {} \sum _{l\in N\backslash \left\{ j,k,m\right\} }R_{\ell }\left( A^{jm,x},E\right) +R_{k}\left( A^{jm,x},E\right) . \end{aligned}$$

Then, $R_{m}\left( A^{jk,x},E\right) =R_{k}\left( A^{jm,x},E\right)$. Hence, $f\left( m,x,E\right) =f\left( k,x,E\right) .$ We then denote it as $f\left( x,E\right) .$ By (1),

$$\begin{aligned} f\left( x,E\right) =\frac{-E}{n-2}. \end{aligned}$$

Notice that

$$\begin{aligned} \left( \left( A^{*}\right) ^{N_{2},\varnothing },\frac{\left| \left| A^{*}\left( N_{2}\right) \right| \right| }{\left| \left| A\right| \right| }E\right) =\left( A^{i^{1}i^{2},\left| \left| A\right| \right| -\alpha _{i}\left( A\right) },\frac{\left| \left| A\right| \right| -\alpha _{i}\left( A\right) }{\left| \left| A\right| \right| }E\right) . \end{aligned}$$

Thus, for each $k\in N\backslash \left\{ i^{1},i^{2}\right\}$,

$$\begin{aligned} R_{k}\left( \left( A^{*}\right) ^{N_{2},\varnothing },\frac{\left| \left| A^{*}\left( N_{2}\right) \right| \right| }{\left| \left| A\right| \right| }E\right) =-\frac{\left| \left| A\right| \right| -\alpha _{i}\left( A\right) }{\left( n-2\right) \left| \left| A\right| \right| }E. \end{aligned}$$

By *essential team on non-cancelled games*,

$$\begin{aligned} R_{i^{1}}\left( \left( A^{*}\right) ^{N_{2},\varnothing },\frac{ \left| \left| A^{1}\left( N_{2}\right) \right| \right| }{ \left| \left| A\right| \right| }E\right) =R_{i^{2}}\left( \left( A^{*}\right) ^{N_{2},\varnothing },\frac{\left| \left| A^{1}\left( N_{2}\right) \right| \right| }{\left| \left| A\right| \right| }E\right) =\frac{\left| \left| A\right| \right| -\alpha _{i}\left( A\right) }{\left| \left| A\right| \right| }E. \end{aligned}$$

We now conclude as follows.

Suppose first that $\alpha _{i}\left( A\right) =0.$ Then, by cases 1.1 and 2.2,

$$\begin{aligned} R_{i}\left( A,E\right) =-\frac{\left| \left| A\right| \right| }{\left( n-2\right) \left| \left| A\right| \right| }E=CD_{i}^{z}\left( A,E\right) . \end{aligned}$$

Suppose now that $0<\alpha _{i}\left( A\right) <\left| \left| A\right| \right| .$ Then, by cases 1.2 and 2.2,

$$\begin{aligned} R_{i}\left( A,E\right)= & {} \frac{\alpha _{i}\left( A\right) }{\left| \left| A\right| \right| }E-\frac{\left| \left| A\right| \right| -\alpha _{i}\left( A\right) }{\left( n-2\right) \left| \left| A\right| \right| }E \\= & {} \frac{\left( n-1\right) \alpha _{i}\left( A\right) -\left| \left| A\right| \right| }{\left( n-2\right) \left| \left| A\right| \right| }E=CD_{i}^{z}\left( A,E\right) . \end{aligned}$$

Suppose finally that $\alpha _{i}\left( A\right) =\left| \left| A\right| \right| .$ Then, by cases 1.2 and 2.1,

$$\begin{aligned} R_{i}\left( A,E\right) =\frac{\alpha _{i}\left( A\right) }{\left| \left| A\right| \right| }E=E=CD_{i}^{z}\left( A,E\right) .\qquad \square \end{aligned}$$

### Proof of Theorem 4

We can prove that $CD^{\ell }$ satisfies *leg-baseline monotonicity* similarly to the case of $ES^{\ell }$ in the proof of Theorem 2. It is straightforward to show that $CD^{\ell }$ satisfies the rest of the axioms in the statement.

Conversely, let *R* be a rule satisfying those axioms. Let $\left( A,E\right) \in {\mathcal {P}}$ be such that $\left| \left| A\right| \right| >0$. Otherwise, the proof is trivial as $R_{i}\left( A,E\right) =\frac{E}{n}$, for each $i\in N$.

Let $m\left( A\right)$ be the number of cancelled games in tournament *A*. We prove that $R\left( A,E\right) =CD^{\ell }\left( A,E\right)$ by induction on $m\left( A\right) .$

Assume first that $m\left( A\right) =0$. Let $i\in N$. Let $A^{*},$ $i^{0},$ $i^{1},$ $i^{2},$ $N_{1}$ and $N_{2}$ be defined as in the proof of Theorem 2. By *weak reallocation proofness*, replicating the argument therein, we obtain that $R_{i}\left( A,E\right) =R_{i}\left( A^{*},E\right)$. Now, as $m\left( A^{*}\right) =0$, $A^{*}=\left( A^{*}\right) ^{z}=\left( A^{*}\right) ^{\ell }.$ This implies that *leg-multi-conference* is equivalent to *zero-multi-conference* for $A^{*}$. Besides, *reallocation proofness* is equivalent to *weak reallocation proofness* for $A^{*}.$ Thus, similarly to the proof of Theorem 3, we can show $R_{i}\left( A^{*},E\right) =CD_{i}^{z}\left( A,E\right) .$ As $m\left( A\right) =0$, $A^{z}=A^{\ell }$. Hence, $R_{i}\left( A^{*},E\right) =CD_{i}^{\ell }\left( A,E\right) .$

Assume then that $R\left( A,E\right) =CD^{\ell }\left( A,E\right)$ for $m\left( A\right) =p$. Let $\left( A,E\right)$ be such that $m\left( A\right) =p+1.$ Then, using similar arguments to those used in the proof of Theorem 2, we can deduce that $R\left( A,E\right) =CD^{\ell }\left( A,E\right) .$ $\square$

### Proof of Theorem 5

It is similar to the proofs of Theorem 1 and Theorem 3 and, thus, we omit it.

## Footnotes

1 See, for instance, https://www.espn.com/olympics/story/_/id/28824781/list-sporting-events-canceled-coronavirus. Last accessed, September, 2022.

2 Csató (2021a) also recently analyzed cancelled sports seasons, but focussing on obtaining a fair ranking. Earlier, Bozóki et al. (2016) analyzed incomplete pairwise comparison matrices to rank tennis players.

3 See also Bergantiños and Moreno-Ternero (2023b).

4 The main national football leagues according to the UEFA coefficient (England, France, Italy, Germany and Spain) follow this format. We should nevertheless acknowledge that some European countries (Belgium, Hungary, and Switzerland among others) have recently employed other tournament formats, which typically include additional round-robin stages (e.g. Lasek and Gagolewski, 2018). Our model could be extended to address for those formats too.

5 In Bergantiños and Moreno-Ternero (2020a) it is assumed that each viewer pays a constant (pay-per-view) fee, which is normalized to 1. Thus, the endowment is not a prior of the model therein as allocating the revenue from broadcasting is the same as allocating the audiences.

6 The domain in Bergantiños and Moreno-Ternero (2020a) is precisely the subset of ${\mathcal {P}}^{c}$, for which the endowment coincides with the aggregate audience of the season. Namely, $\left( A,E\right) \in {\mathcal {P}}^{c}$ such that $E=||A||$.

7 And also used in Bergantiños and Moreno-Ternero (2020b, 2021, 2022a, 2022b, 2022c, 2022d, 2023a, 2023b).

8 The concept of operators on the space of allocation rules is explored in detail by Thomson and Yeh (2008) and Thomson (2019). See also Hougaard et al. (2012) and Moreno-Ternero and Vidal-Puga (2021).

9 This axiom and the next one are natural counterparts of those introduced in Bergantiños and Moreno-Ternero (2020a) for the benchmark setting.

10 *Monotonicity* is a general principle of fair division which states that when the underlying data of a problem change in a specific way, the solution should change accordingly. Early formalizations of this principle, for somewhat related models, can be traced back to Meggido (1974), Kalai and Smorodinsky (1975), Thomson and Myerson (1980), or Young (1985), among others. It has also been recently used in other fields such as sports rankings and tournament design (Csató , 2020, 2021b, 2022a, b).

11 This is reminiscent of the concept of baselines in rationing problems formalized by Hougaard et al. (2012, 2013a, 2013b). See also Ju et al. (2021).

12 Baseline monotonicity can be strengthened naturally with strict inequalities. But our characterization results would not be altered with the ensuing (stronger) axioms.

13 Ju et al. (2007) analyze the implications of this kind of axioms in general allocation problems.

14 This is the case, for instance, of the four major sports leagues in North America.
